# Evaluation of a Scintillating Plastic Optical Fiber Device for Measuring kV-Cone Beam Computed Tomography Dose

**DOI:** 10.3390/s23187778

**Published:** 2023-09-09

**Authors:** Christian Popotte, Romain Letellier, Didier Paul, Alexandre Waltener, Nicolas Guillochon, Mélodie Munier, Paul Retif

**Affiliations:** 1 INSERM Unité U1296 Radiations: Défense, Santé Environnement, 69008 Lyon, France; 2 Fibermetrix, 7 Allée de l’Europe, 67960 Entzheim, France; 3 Medical Physics Unit, CHR Metz-Thionville, 57000 Metz, France; 4 Centre National de la Recherche Scientifique, Centre de Recherche en Automatique de Nancy, Université de Lorraine, 54000 Nancy, France

**Keywords:** CBCT, IGRT, scintillating dosimetry, optical fiber

## Abstract

Background: Justification of imaging procedures such as cone beam computed tomography (CBCT) in radiotherapy makes no doubt. However, the CBCT composite dose is rarely reported or optimized, even though the repeated CBCT cumulative dose can be up to 3% of the prescription dose. This study aimed to evaluate the performance and utility of a new plastic scintillating optical fiber dosimeter for CBCT dosimetric quality assurance (QA) applications before a potential application in patient composite CBCT dosimetry. Methods: The dosimeter, made of 1 mm diameter plastic fiber, was installed under a linear accelerator treatment table and linked to photodetectors. The fiber impact on the fluence and dose delivered was respectively assessed with an electronic portal imaging device (EPID) and EBT3 Gafchromic^®^ film. The presence of artifacts was visually evaluated on kV images. The dosimeter performances were determined for various acquisition parameters by comparison with ionization chamber values. Results: The maximum impact of the fiber on the fluence measured by the EPID was −1.2% for the 6 MV flattening filter-free beam. However, the fiber did not alter the film dose profile when measured for all the beams tested. The fiber was not visible at energies ≥ 80 kV and was merely visible on the CBCT images. When the rate of images per second or mA was changed, the maximum relative difference between the device and the ionization chamber CTDIs was <5%. Changing collimation led to a −7.2% maximum relative difference with an absolute dose difference that was insignificant (−0.3 mGy). Changing kV was associated with a −8.7% maximum relative difference, as published in the literature. Conclusions: The dosimeter may be a promising device for CBCT recurrent dosimetry quality control or dose optimization. According to these results, further developments are in progress in order to adapt the solution to the measurement of patient composite CBCT doses.

## 1. Introduction

Modern radiotherapy is increasingly using modulated dose delivery techniques to deliver radiation doses to multiple body areas with a high degree of conformity. However, the dose distribution at each fraction can vary according to patient-related variables, namely, changes in patient anatomy, movement during treatment, and variations in patient setup. Since these variations can have a significant effect on radiotherapy precision [1,2,3], it is vital to verify the patient setup at each treatment fraction. To address this, kV-cone beam computed tomography (CBCT) was devised in the early 2000s to periodically visualize the volumetric patient anatomy during treatment. Although higher doses are needed compared to traditional two-dimensional kV or MV imaging, the benefits of CBCT mean that it is increasingly being used in radiotherapy. Indeed, CBCT imaging is now available in the radiation-delivery units of different vendors, including Varian-Siemens Healthineers^®^ Company (Palo Alto, CA, USA) and Elekta AB (Stockholm, Sweden). More recently, accelerators such as Radixact^®^ (Accuray Incorporated; Sunnyvale, CA, USA) have also been devised to acquire computed tomography (CT) images during treatment; these accelerators have the advantage that they can potentially acquire longer longitudinal fields of view. However, they, too, require higher doses than two-dimensional kV or MV imaging.

Since the dose resulting from imaging in radiotherapy is two orders of magnitude smaller than the therapeutic doses (1–10 cGy per scan) [4,5,6,7,8,9,10,11], it is often not considered in the planning process and is rarely reported at the end of the treatment. However, the cumulative dose when repeated scans are conducted can be significant. For example, the cumulative dose of kV-CBCT procedures for pelvic imaging can be 1–3% of the prescription dose (e.g., up to 2.3 Gy for 78-Gy prostate treatment) [12]. Moreover, the body area that is irradiated during an imaging procedure is often larger than the treatment field, which results in unintended irradiation of nearby organs and an increased risk of radiation-induced pathologies [13,14,15,16,17,18].

Radiotherapy centers vary markedly in terms of volumetric-imaging protocols, including the frequency of CBCT and the acquisition parameters. For example, some centers use daily volumetric images, whereas others acquire these images daily during the first week and then once a week to check that the patient is following the nutritional guidelines or to determine whether new treatment planning is needed [19]. Notably, each vendor also has its own acquisition protocol, which is generally related to the body part, the image quality, or the field of view. These protocols can be used without alteration or can be optimized by the local medical physics team but can also significantly shape the cumulative dose received by the patient during imaging. Therefore, it has been recommended by multiple institutions [20,21,22] to (i) work towards an easy reporting of the imaging dose given through radiotherapy treatment courses and (ii) implement quality-control systems that check the X-ray tube dosimetric and image quality performances.

To address these issues, Fibermetrix^®^ (Entzheim, France) has developed an innovative device that can measure the imaging dose that is delivered by kV-CBCT acquisition and automatically recalculate the corresponding dose metric for QA applications. Since the computed tomography dose index (CTDI) is the most commonly applied dose quantity in CT and CBCT dosimetry, even though this index is insufficient for composite patient dosimetry, the device was first validated for QA applications using the CTDI dosimetric quantity. Given that the actual spatial distribution of the imaging dose to the patient (and even the average) can differ substantially from the CTDI value, the possibility of using the detector to measure the composite image-guided radiotherapy (IGRT) dose should be assessed in further work using different dose quantities than the one used for QA applications. The device was derived from an original device developed for CT applications [23,24,25], named IVIscan^®^. We recently adapted this device to radiotherapy kV-CBCT applications. The detector, namely IVI-CBCT, is based on a plastic optical fiber (POF) and a plastic scintillating fiber (PSF) dosimeter attached under the treatment couch. Here, we describe the device, its negligible effect on the treatment dose and delivered CBCT dose and imaging, and its accuracy compared to the pencil-chamber reference for various CBCT acquisition parameters.

## 2. Materials and Methods

### 2.1. Properties of the Dosimeter

**Figure 1:**
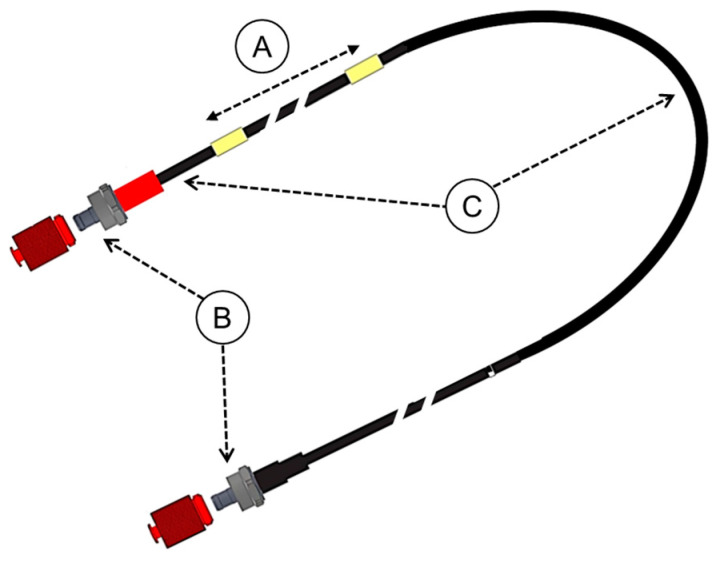
Drawing of the IVI-CBCT probe. (**A**) The PSF. (**B**) The connectors. (**C**) The clear POF.

The dosimeter developed here is composed of two photodetectors, each of which is connected to an exit extremity of a U-shaped 1 mm diameter optical fiber probe. The probe consists of a 1 m long BCF12 PSF (Saint-Gobain, Courbevoie, France) and clear POF acting as a light guide (Figure 1). The PSF bears a polystyrene-based core clad with poly(methyl methacrylate) (PMMA) and has a density of 1.05 g/cm^3^, which makes the dose measurements free of density corrections for the dose to water. To guarantee that the probe efficiently traps the emitted photons, the core and cladding of the PSF have a refractive index of 1.6 and 1.49, respectively. In order to ensure the light isolation of the fiber and guarantee the mechanical resistance of the probe, its entire length is surrounded by a 3 mm outer-diameter cladding made of black Hytrel^®^. The photodetectors are each composed of a photosensitive surface that transforms visible light into electrons and a photomultiplier. They are connected to a discriminator that suppresses noise in the signal. The total number of photons collected by the dosimeter is called the count rate. The dosimeter is designed to measure doses between 1 μGy and 1.8 kGy (uncertainty < 1%; resolution 0.02 nGy) with dose rates ranging between 1 μGy/s and 250 mGy/s (uncertainty < 1%; resolution 0.02 nGy/s). Its temporal resolution is <1 ms, and its energy dependency was found by testing to be 1% in the 70–150 kV range for the following standard radiology beam qualities: RQT, RQR, RQA, and N [26]. Its angular dependency is <3%. The dosimeter is connected to a wall-mounted receiver via Bluetooth. The latter is connected to a computer hosting the analysis software via an RJ45 cable.

### 2.2. Installation of the Device on the Irradiation Device

**Figure 2:**
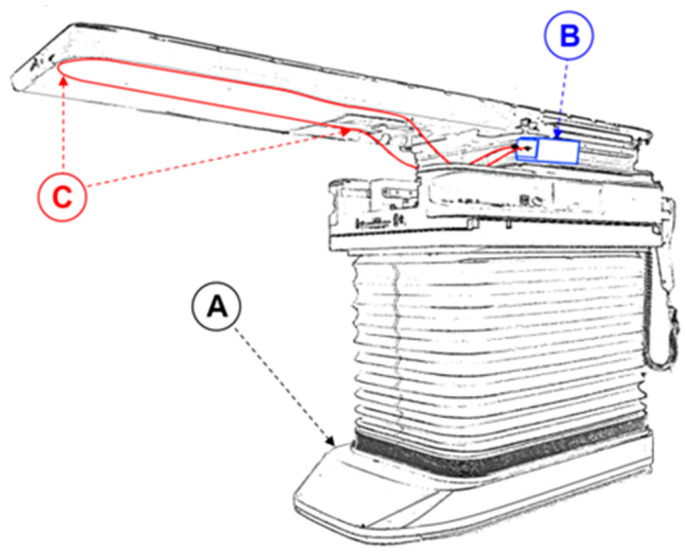
Schematic depiction of the components of the measurement device and its setup on the treatment couch. (**A**) The foot of the couch of the accelerator. (**B**) The signal treatment unit composed of a battery, two photomultipliers, a signal analysis controller, and a Bluetooth emitter. (**C**) The optical fiber, which is securely taped beneath the couch top. The two arrows point to the beginning and the end of the scintillating part of the fiber.

To test the utility of the dosimeter, it was installed on the PerfectPitch Exact Couch of a Varian TrueBeam STx accelerator (Varian-Siemens Healthineers Company; Palo Alto, CA, USA) at Metz-Thionville Regional Hospital (Metz, France). The IVI-CBCT was affixed with tape under the couch top such that its scintillating part was aligned along the longitudinal midline of the top. It was then connected to the amplification system, which was attached to the side of the couch (Figure 2). The accelerator was equipped with an onboard kV imager capable of acquiring planar kV images as well as kV-CBCT images.

### 2.3. Calibration of the Device for Specific CBCT Protocols

Before use, the dosimeter was cross-calibrated for each CBCT protocol by employing a pencil ionization chamber that had been calibrated in a primary standards laboratory. Thus, the cross-calibration factor Nc is determined for each protocol by conducting reference acquisitions. Nc is defined as follows:

N_c, protocol_ = CTDI_ref,IC_/M_ref,IVI-CBCT_ (1)

Here, CTDI_ref,IC_ was measured during the reference acquisitions using a 10 cm PTW (PTW-Freiburg, Freiburg, Germany) pencil IC model 30009 connected to a PTW DIADOS E model T11035 electrometer and placed in a dedicated PTW model T40027 CT body phantom using the methodology developed earlier in the thesis [25] based on the IAEA HUMAN HEALTH REPORTS No. 5 methodology [21], and M_ref,IVI-CBCT_ is the integrated number of counts measured by the IVI-CBCT device during the reference acquisitions.

Concerning the CTDI measurements, a reference supero-inferior collimation of 2 cm and a nominal beam width of 17.5 or 18.5 cm, depending on the CBCT protocol, was used with the following formula:

(2) $${CTDI}_{100,nominal\;width}={CTDI}_{100,20}\times \frac{{CTDI}_{free-in-air,nominal\;width}}{{CTDI}_{free-in-air,20}}$$

where CTDI_100,nominal width_ is the CTDI calculated in the phantom for the nominal beam width; CTDI_100,20_ is the CTDI measured in the phantom for the reference beam width (20 mm); CTDI_free-in-air nominal width_ is the CTDI measured in the air for the nominal beam width at 3 incremented supero-inferior positions, −10 cm, 0 cm, and +10 cm from the isocenter; and CTDI_free-in-air, 20_ is the CTDI measured in the air for the reference beam width.

The CTDI is then determined for each acquisition using the following formula:

CTDI_IVI-CBCT_ = N_c,protocol_ × M_IVI-CBCT_ (3)

where CTDI_IVI-CBCT_ is the CTDI measured with the IVI-CBCT system and M_IVI-CBCT_ is the integrated number of counts measured by the IVI-CBCT device during the acquisition.

**Figure 3:**
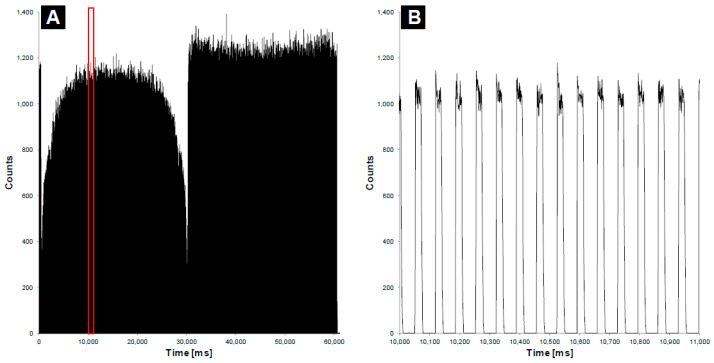
Counts as a function of time (ms) during a CBCT acquisition with 360° rotation of the source (**A**) and a zoomed view of (**A**) inside the red boundaries showing the pulsed emission of X-rays from the kV source (**B**).

All the calibrations and formulas mentioned above are integrated into the pre-commercial analysis software (see Figure 3) developed specifically for the fiber dosimeter measurement system. The software was developed using C# and installed on a computer running on Windows present at the control desk. The communication with the dosimeter was assured using an RJ45 cable through the bunker’s cable conduits. The software allowed the operators to register a calibration factor for each CBCT protocol used (see Figure 3). Depending on the time between two CBCT-emitted pulses (see Figure 3) and the probe’s dark count rate (DCR) noise, the user could also determine other signal processing parameters, like a cutting parameter between two pulses or a DCR threshold parameter. The count rate during the acquisition was displayed on the readout screen, as shown in Figure 3.

### 2.4. Determining the Impact of the Fiber on the Delivery of the Treatment Dose and Imaging

We conducted two tests to determine whether the physical presence of the fiber dosimeter could modify the delivery of the treatment and two tests to assess the effect of the fiber on 2- or 3-dimensional images.

**Figure 4:**
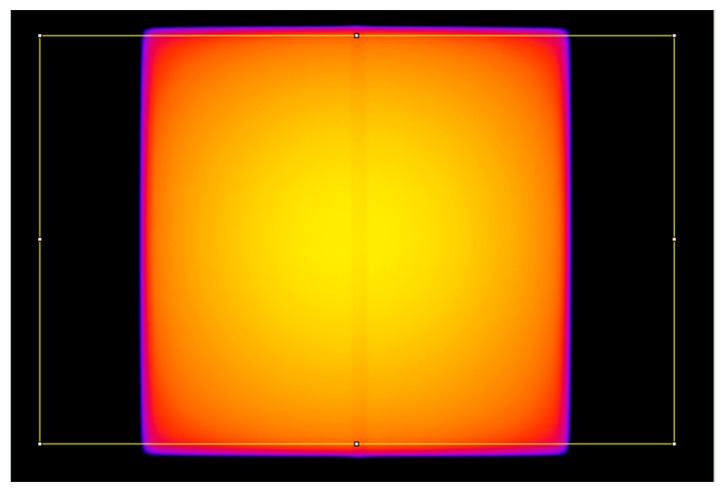
Example of a 10 cm × 10 cm 6 MV FFF fluence image acquired with the electronic portal imaging device (EPID) in the presence of the fiber with a 10 cm thick RW3 phantom at the isocenter. The figure shows the rectangular region of interest (ROI) that was used to compute average profiles in the left/right direction to assess the impact of the dosimeter.

First, we acquired posterior 10 cm × 10 cm 6 MV Flattening Filter-Free (FFF), 6 MV with flattening filter, and 18 MV fluence [27] images with the electronic portal imaging device (EPID) of the TrueBeam both with and without the device. During these measurements, a 10 cm thick RW3 slab phantom (PTW-Freiburg^®^, Freiburg, Germany) was placed on the table at the isocenter in order to reproduce a beam going through a volume with a density close to water. The Digital Imaging and Communications in Medicine standard (DICOM standard) images were analyzed using the open-source image-processing program ImageJ version 1.53v [28]; specifically, a large region of interest (ROI) encompassing the majority of the beam was used to compute an average profile in the left/right direction (Figure 4). The images with and without the fiber were then compared in terms of normalized intensity.

Second, we placed an EBT3 Gafchromic^®^ film (Ashland Advanced Materials, Bridgewater, NJ, USA) at the isocenter in the middle of a 10 cm RW3 slab phantom to reproduce a measurement inside a volume of density close to the patient and irradiated the film with a 2 Gy dose with a posterior 10 cm × 10 cm 6 MV FFF beam. The film was scanned before and after irradiation with an Epson^®^ Expression 10000XL scanner (Seiko Epson Corp.; Suwa, Japan). The dose analysis was performed with in-house Java software version 8, in which 3-color conversion and film homogeneity correction were implemented by using the non-irradiated image. The analysis was conducted using the same methodology as described above for the EPID images, except that the reference profile (i.e., without the dosimeter) was calculated by the Varian Eclipse 16.1 Treatment Planning System (TPS) (Varian Medical Systems, Palo Alto, CA, USA) using the Accuros XB 15.6.04 algorithm with a 1 mm calculation grid size and the same geometry.

Third, the probe visibility and potential presence of artifacts were evaluated. Posterior kV images were acquired in the presence of the fiber without and with an in-house 3D-printed thoracic phantom by using two acquisition protocols, namely, “Extremity” with 65 kV and 3.5 mAs and “Thorax Small” with 80 kV and 5.0 mAs. The images were assessed for the visible presence of the fiber. Finally, the CBCT image sets acquired in the presence of the fiber without a phantom using the “Head Full Fan” protocol with 100 kV and 270 mAs were assessed visually.

### 2.5. Comparison of the Device, Pencil Ionization Chamber, and Accelerator CTDIs

The CTDI was measured with the IVI-CBCT device for three CBCT protocols that are detailed in Table 1. The measurements were taken in the air with the fiber at the isocenter; the couch’s vertical axis was +5.4 cm. These CTDIs were compared to the pencil chamber CTDI measurements for these protocols.

Repeatability was assessed by taking 10 CTDI measurements for each protocol and each detector. The mean value and standard deviation of these measurements were recorded, and the coefficient of variation was calculated. The CTDI value reported by the accelerator was also recorded and compared to the mean device and pencil-chamber values.

How the device CTDI varied when several acquisition parameters of the Pelvis protocol were changed was also assessed with both detectors. These acquisition parameters were the tube voltage (100 or 125 kV), tube current (20, 60, or 100 mA), collimation (2, 4, 10, 15, or 17.5 cm), and images per second (IPS) (3 or 15 IPS).

The minimum time required to realize the CTDI measurement by each detector to measure the CTDI was also evaluated.

## 3. Results

### 3.1. Determining the Impact of the Fiber on the Delivery of the Treatment Dose

**Figure 5:**
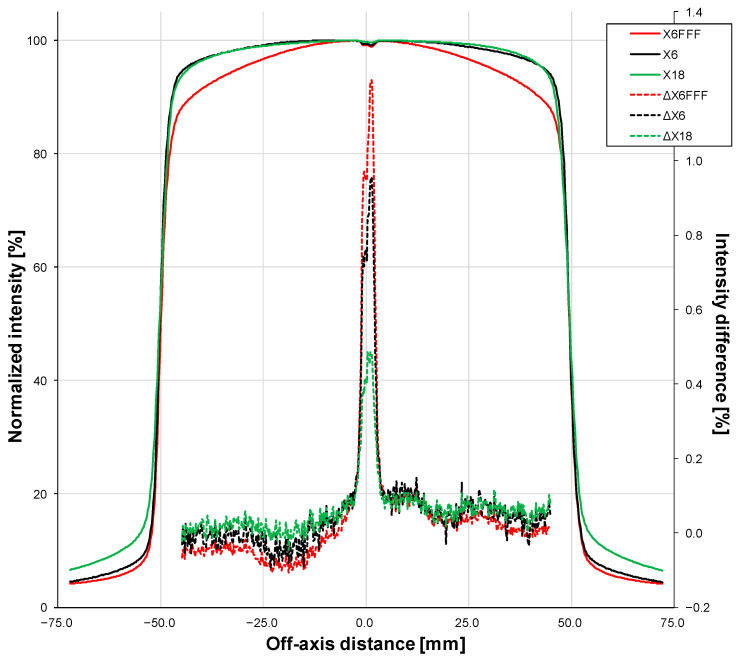
Normalized intensity profiles (solid lines, left vertical axis) of 10 cm × 10 cm 6 MV FFF, 6 MV, and 18 MV fluence images acquired with the electronic portal imaging device (EPID) in the presence of the fiber with a 10 cm thick RW3 phantom at the isocenter. The absolute differences between the profiles when the fiber was absent are also shown (dashed lines, right vertical axis).

We first conducted four tests to determine whether the presence of the device affected the delivery of the treatment or the imaging. In the first, fluence profiles of three beam energies (6 MV FFF, 6 MV, and 18 MV) were acquired with EPID in the presence or absence of the fiber with a 10 cm thick RW3 phantom at the isocenter. The fluence profile of each beam energy in the presence of the fiber is shown in Figure 5 (solid lines). Absolute differences between the profiles when the fiber was absent are also shown (dashed lines). For all three beam energies, the presence of the fiber decreased the signal in the center of the profile (i.e., between −2 mm and +2 mm). The drop was greatest for the 6 MV FFF beam (maximum −1.2%), followed by the 6 MV beam (maximum −1.0%) and then the 18 MV beam (maximum −0.5%).

**Figure 6:**
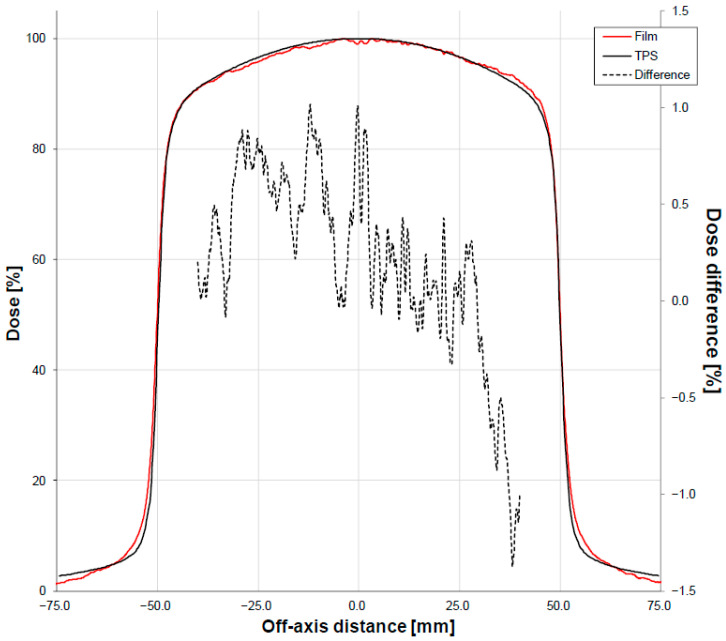
Dose profile (solid lines, left vertical axis) of the 10 cm × 10 cm 6 MV FFF beam energy obtained from an EBT3 film placed at the isocenter in the middle of a 10 cm thick RW3 phantom in the presence of the fiber and the corresponding dose calculated using the TPS Eclipse. The absolute differences between these doses are also shown (dashed line, right vertical axis).

The second test involved placing an EBT3 film at the isocenter in the middle of a 10 cm thick RW3 phantom in the presence of the fiber, applying the 6 MV FFF beam, determining the dose profile from the film, and comparing it to the dose profile calculated using the TPS Eclipse. No relevant differences between the film and the TPS can be seen, even in the middle of the profiles where the fiber was located (Figure 6).

### 3.2. Determining the Impact of the Fiber on the Delivery of the Imaging

The third test was to compare the kV images that were acquired with the “Extremity” (65 kV and 3.5 mAs) and “Thorax Small” (80 kV and 5.0 mAs) protocols in the presence of the fiber with and without a thoracic phantom.

**Figure 7:**
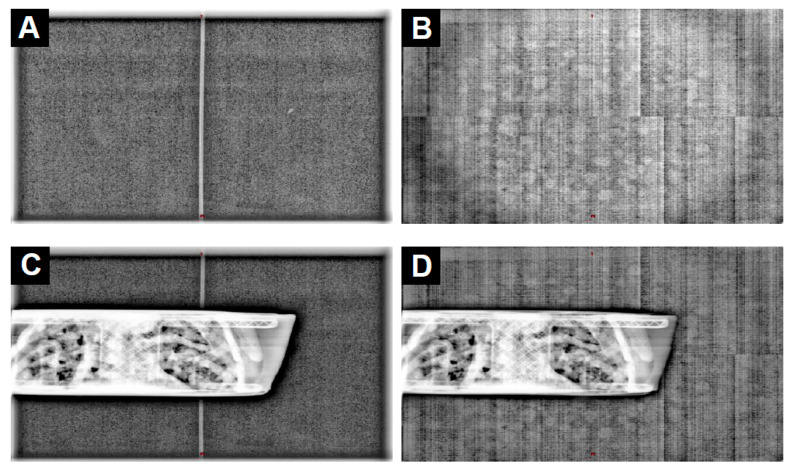
Planar images acquired using a posterior incidence without (**A**,**B**) and with (**C**,**D**) an in-house 3D-printed thoracic phantom using the “Extremity” (65 kV and 3.5 mAs; (**A**,**C**)) and “Thorax Small” (80 kV and 5.0 mAs; (**B**,**D**)) acquisition protocols.

When the phantom was not present, the fiber was visible in the middle (left/right direction) of the kV images, but only when they were acquired at 65 kV (Figure 7A); the fiber was not visible at 80 kV (Figure 7B). Moreover, the fiber could not be seen on the pixels covered by the thoracic phantom (Figure 7C). Notably, while appearing on low-kV images (65 kV) without fantom, the fiber did not generate artifacts on the images when used with a fantom or at 80 kV.

**Figure 8:**
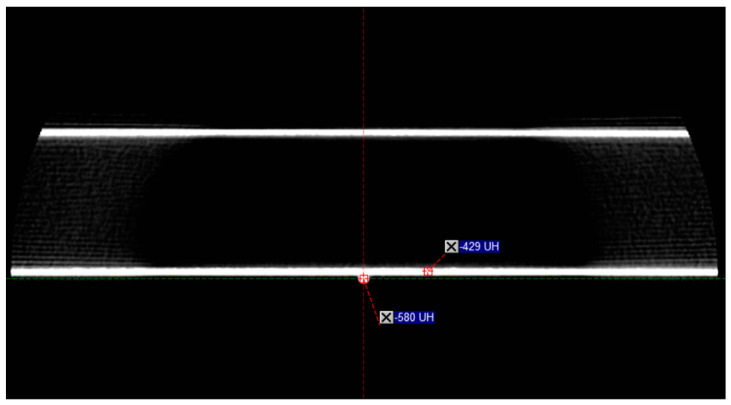
An axial image of a CBCT image set without phantom acquired using the “Head Full Fan” protocol with 100 kV and 270 mAs. The carbon treatment couch appears as two horizontal white lines, and the fiber as a white point beneath the couch. Two point measurements are displayed: one in the lower edge of the couch and one in the fiber.

The fourth test involved visually assessing axial CBCT images acquired in the presence of the fiber without a phantom using the “Head Full Fan” protocol with 100 kV and 270 mAs. Figure 8 shows the carbon treatment couch (two horizontal white lines) with an example of a point measurement (−429 Hounsfield Units (HU)) and the fiber beneath the couch with another point measurement (−580 HU).

The HU value of the fiber was close to the treatment couch, and the presence of the fiber did not interfere with the treatment couch image or HU value.

### 3.3. Comparison of the Device, Pencil Ionization Chamber, and Accelerator CTDIs

**Figure 9:**
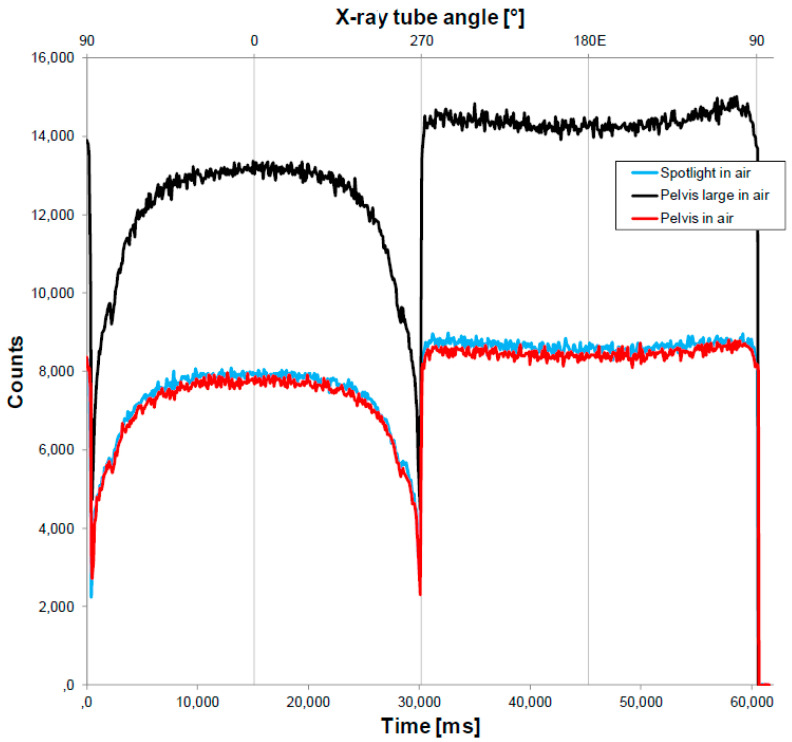
Number of counts measured in air by the device as a function of time (ms) and the X-ray tube angle (°) for the three CBCT protocols (Pelvis, Pelvis Large, and Spotlight).

The device was then tested in terms of how reliably it measured the CTDI with three CBCT protocols termed Pelvis, Pelvis Large, and Spotlight (Table 1). Figure 9 shows the counts over time that were measured in air using the fiber dosimeter. The shapes of the acquisition curves are typical of a full rotation of the gantry. Specifically, at the beginning (first seconds, gantry at 180°, X-ray tube at 90°), the signal drops drastically because the kV beam runs into the edges of the couch; it then rises until a maximum is reached when the X-ray tube is at 0° (gantry at 90°); subsequently, it drops again when crossing the other side of the table. Each CBCT protocol was tested 10 times using the IVI-CBCT device and the IAEA method with the pencil chamber. The standard deviation and coefficient of variation of the 10 iterations were calculated. Table 2 shows the mean, standard deviation, and coefficient of variation of the 10 iterations. The values reported by the linear accelerator are also shown. Table 2 presents the mean and standard deviation values graphically. The CTDIs ranged from 19 to 45 mGy for the three protocols, and the mean device CTDIs were almost identical to the mean pencil-chamber CTDIs for all three. Moreover, the device displayed better consistency than the pencil chamber, as shown by the coefficients of variation (Pelvis: 0.1% vs. 1.7%; Pelvis Large: 0.1% vs. 0.5%; Spotlight: 0.5% vs. 0.5%). Notably, the CTDIs reported by the accelerator were persistently lower by 10–17% depending on the protocol.

**Figure 10:**
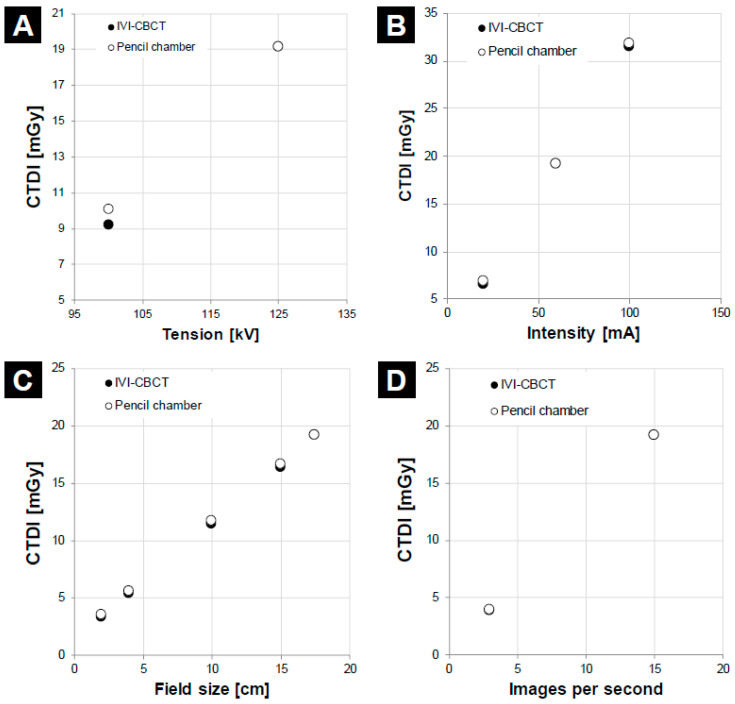
Plots showing the CTDI in mGy measured by the IVI-CBCT dosimeter and the pencil chamber when the Pelvis protocol was used, but the tube voltage [kV] (**A**), tube current [mA] (**B**), field size [cm] (**C**), or number of images per second (**D**) was altered.

Finally, we tested the device by varying the acquisition parameters of the Pelvis CBCT protocol, namely, tube voltage (100 or 125 kV), tube current (20, 60, or 100 mA), collimation (2, 4, 10, 15, or 17.5 cm), and IPS (3 or 15). Figure 10 compares the CTDIs that were calculated using the device and the pencil chamber. Close agreement was generally obtained: the maximum relative differences for tube voltage, tube current, collimation, and IPS were, respectively, −8.7% (at 100 kV), −4.6% (at 20 mA), −7.2% (at 15 cm), and−2.6% (at 3 IPS).

## 4. Discussion

Our tests of the device started with analyses of the impact of IVI-CBCT on the dose that was delivered. We found that the EPID signal changed at the exact position of the fiber beneath the couch: the maximum difference was −1.2%, which was observed with the 6 MV FFF beam (Figure 5). This change could be caused by the known over-sensitivity of amorphous silicon EPIDs to lower energy photons compared to water-equivalent detectors; this was due to the increased photoelectric effect in the copper/phosphor screen [29,30,31]. The notion that the EPID signal change that we observed was due to EPID’s over-sensitivity to low-energy photons is supported by the fact that the fiber did not affect the dose measurements of the film (Figure 6). Thus, the presence of the fiber does not appear to alter the delivery of the therapeutic dose.

Regarding the images (Figure 8), the probe was visible at low voltage (65 kV) but disappeared from the image at 80 kV. Given that the CBCT protocols present on the linac used all involve a voltage starting at 80 kV, the probe could be considered invisible from the CBCT. Nevertheless, the interference of the probe was not evaluated for all the imaging protocols available on the linac, such as Exactrac (Brainlab, Munich, Germany) or MV-2D images, and may be visible on low-dose imaging protocols using voltage < 80 kV.

Notably, Figure 9 shows how the counts measured by the detector varied over time and, therefore, the X-ray source angle. The measured signal was highly dependent on the X-ray tube position and, therefore, on the treatment couch attenuation. Thus, this perspective was not investigated during this study, but the shape of the signal could be used to assess, for example, the position of the fiber beneath the table or any modification in the tube rotation geometry.

During this study, we cross-calibrated the IVI-CBCT dosimeter with a pencil chamber that had itself been calibrated in a primary standards laboratory. To test the accuracy with which the device measured CTDI, we repeated this cross-calibration for three acquisition CBCT protocols (Pelvis, Pelvis Large, and Spotlight) that require different kV adjustments (125 or 140 kV) and fans (half fan or full fan); these protocols were chosen because our previous evaluations of the detector with CT scanners showed a non-negligible dependence of the response of the fiber on the energy of the beam; specifically, we found up to 31% of dose deviations between a scintillating fiber dosimeter and a pencil ionization chamber in a previous study [24]. Thus, we acquired 10 measurements for each detector and each protocol and found that IVI-CBCT demonstrated better constancy (coefficient of variation ranged from 0.1% to 0.5%) than the pencil chamber (0.5% to 1.7%). Notably, compared to the CTDIs measured using the device or pencil chamber, the CTDIs reported by the accelerator were consistently lower. This could be attributed to the dose-estimation methodology of the accelerator. Our analyses with the three CBCT protocols also showed that the dose involved in the imaging ranges from 19 to 45 mGy, depending on the protocol. Since patients can undergo several CBCTs at each treatment fraction, this finding supports a previous study showing that the overall dose from kV-CBCT imaging can reach 3 Gy by the end of the treatment.

We also tested the accuracy of the device when the acquisition parameters in one of the CBCT protocols (Pelvis) were altered. Again, the IVI-CBCT dosimeter agreed well with the pencil chamber. Specifically, the relative differences were <5% when changing the IPS from 3 to 15 or the mA from 20 to 60 or 100. As expected, because of previous studies on scintillating fiber dosimeters [24], when dropping the tube voltage from 125 to 100 kV, the relative dose difference was −8.7%. Notably, when varying the field size, the maximum relative dose difference was −7.2%, which occurred at a field size of 2 cm. However, it is not likely to be a major concern because the absolute dose difference was very low (−0.3 mGy). On average, the trends in the variation of the responses of the pencil ionization chamber and the scintillating fiber dosimeter with dose parameters are the same, showing that the IVI-CBCT can be used to detect variations in the tube output over time.

Finally, it has to be noted that the developed dosimeter, as opposed to the ionization chamber, requires a unique measurement, while the IC requires multiple measurements to assess the CTDI. This results in a minimum requested time of 60 s (time of a CBCT acquisition) to measure the CTDI with the IVI-CBCT dosimeter, whereas the pencil IC requests a minimum of 540 s to perform the same measurement. Because of its design, the device generates the CTDI very quickly, namely, in the time needed to conduct a CBCT acquisition. This is considerably faster than the reference IAEA method, which takes at least nine times this duration for a complete set of acquisitions. This reflects the fact that these latter measurements must be conducted in a dedicated phantom and free-in-air and that great care must be paid to the ionization chamber position. Specifically, the phantom must be positioned, and measurements in the five holes and free-in-air must be conducted in three positions. In addition, the medical physicist’s time in radiotherapy is often spread across numerous tasks, resulting in the need for fast and accurate tools. In this context, the device presented in this thesis presents a significant advantage in terms of the timeliness of providing the CTDI.

Given the increase of CBCT acquisitions in IGRT, it is possible that the CBCT dosimetric quality controls become more recurrent in the next years. In this context, the promising properties of IVI-CBCT presented in this article suggest that it can be useful for CTDI verifications (e.g., periodic mandatory controls following an intervention), tube output monitoring over time, and particularly for protocol optimization purposes requesting several dose measurements using various acquisition parameters. However, further studies are needed to determine how well the device performs with a variety of CBCT protocols, including cranial CBCT protocols or optimized low-dose protocols, and other onboard CBCT manufacturers. Moreover, given the efficiency of this methodology with regard to quality control or dose optimization measurements, it is of interest to determine whether it can be used to measure the CBCT doses that are delivered to the patient during radiotherapy treatment. This latter issue also points to the CBCT patient dose metrics topic. In this context, an innovative measurement approach can help delineate the shape of uniform practices and be of great interest to the community.

## 5. Conclusions

Our study suggests that the dosimeter proposed in this paper may be promising for quality control or dose optimization of the CBCT acquisitions on dedicated radiotherapy linear accelerators. Moreover, we demonstrated that the presence of the detector has little to no influence on the MV treatment beams or kV images in either two- or three-dimensional modes. This suggests that the device can also be used for CBCT patient dosimetry applications if it is accompanied by a suitable technique.

## Figures and Tables

**Table 1:** CBCT protocols used for the study.

Protocol Details	Pelvis	Pelvis Large	Spotlight
Fan type	Half fan	Half fan	Full fan
Trajectory	Full	Full	Full
Start angle (°)	180	180	180
End angle (°)	180E ^1^	180E ^1^	180E ^1^
Tube voltage (kV)	125	140	125
mAs	1080	1687.5	1350
Field of view (cm)	46.5	46.5	26.2
Collimation (cm)	17.5	17.5	18.5

^1^ E stands for extended.

**Table 2:** For the three protocols tested, CTDI measured by IVI-CBCT and the pencil chamber and CTDI reported by the accelerator.

Source of CTDI Value		CBCT Protocols	
	Pelvis	Pelvis Large	Spotlight
IVI-CBCT	19.14 ± 0.02 mGy [0.1%]	40.45 ± 0.03 mGy [0.1%]	25.34 ± 0.11 mGy [0.5%]
Pencil chamber	19.14 ± 0.33 mGy [1.7%]	40.45 ± 0.15 mGy [0.5%]	25.34 ± 0.12 mGy [0.5%]
Reported by accelerator	15.89 mGy	36.54 mGy	22.02 mGy

The IVI-CBCT and pencil-chamber data are expressed as mean ± standard deviation mGy [coefficient of variation %].

## Data Availability

The data presented in this study are available on request from the corresponding author.
